# Singular value decomposition-based regression identifies activation of endogenous signaling pathways *in vivo*

**DOI:** 10.1186/gb-2008-9-12-r180

**Published:** 2008-12-18

**Authors:** Zhandong Liu, Min Wang, James V Alvarez, Megan E Bonney, Chien-chung Chen, Celina D'Cruz, Tien-chi Pan, Mahlet G Tadesse, Lewis A Chodosh

**Affiliations:** 1 Department of Cancer Biology, Abramson Family Cancer Research Institute, University of Pennsylvania, 421 Curie Blvd, BRB II/III 616, Philadelphia, PA 19104, USA; 2 Genomics and Computational Biology Graduate Group, University of Pennsylvania School of Medicine, 423 Guardian Drive, Philadelphia, PA 19104, USA; 3 Department of Mathematics, Georgetown University, 2115 G Street NW, Washington, DC 20057, USA

## Abstract

The ability to detect activation of signaling pathways based solely on gene expression data represents an important goal in biological research. We tested the sensitivity of singular value decomposition-based regression by focusing on functional interactions between the Ras and transforming growth factor beta signaling pathways. Our findings demonstrate that this approach is sufficiently sensitive to detect the secondary activation of endogenous signaling pathways as it occurs through crosstalk following ectopic activation of a primary pathway.

## Abstract 2

Singular value decomposition regression can detect the activation of endogenous signaling pathways, allowing the identification of pathway cross-talk.

## Background

Tumors arise following the accumulation of a diverse set of genetic aberrations within a single cell [1]. This heterogeneity makes prognostic and therapeutic decisions difficult, as tumors arising from the same tissue type may harbor activation of distinct oncogenic pathways [2,3]. As a consequence, tumors that are histologically similar may follow strikingly different clinical courses and respond differently to conventional and targeted therapies [4-6]. Indeed, as molecularly targeted therapies increasingly enter the clinic, identifying the spectrum of oncogenic pathways activated within a given tumor will become even more critical for selecting effective therapeutic approaches.

Currently, the clinical detection of oncogenic pathway activation is most commonly performed using methods that analyze pathway activation at the protein level, such as immunohistochemistry to detect oncogene overexpression, or at the DNA level to detect oncogene amplification, with techniques such as fluorescence *in situ *hybridization (FISH) and quantitative PCR. For example, expression of human epidermal growth factor receptor 2 (HER2) and estrogen receptor are routinely assessed to guide treatment selection in breast cancer [7,8]. Unfortunately, many commonly activated oncogenic pathways do not lend themselves to this type of analysis. This is, in part, due to the fact that most pathways can be activated at multiple points in the pathway [3], thereby complicating attempts to assess a pathway's overall activation status. Consequently, a more robust and generalizable method for detecting oncogenic pathway activation in tumors would be valuable.

To date, a number of methods have been developed to infer pathway activation from gene expression data. These approaches have the advantage of being applicable to multiple pathways simultaneously and of requiring only one technological modality. For example, gene set enrichment analysis (GSEA) has been used to detect pathway activation by comparing the extent of enrichment of a signature for a given pathway between two groups of samples [9]. Using this approach, Sweet-Cordero *et al*. [10] detected a K-Ras expression signature in human lung adenocarcinomas bearing K-Ras mutations.

However, GSEA has several limitations. First, it cannot provide a quantitative measure of pathway activation. More importantly, since GSEA relies on a comparison between two groups, it cannot be used to identify the state of pathway activation for individual samples. This represents a major limitation, since separating a sample set into two groups for the purposes of comparison requires prior knowledge of some relevant feature of the samples. Consequently, GSEA is most useful for identifying pathways that are enriched in samples with a known clinical parameter, such as a particular tumor subtype. In contrast, GSEA is not well suited for identifying or comparing pathway activity levels within a group of samples. Other enrichment analysis methods, such as gene set analysis [11], share these shortcomings.

An alternative approach to detecting pathway activation is singular value decomposition-based Bayesian binary regression (SVD regression) [7,12]. In this approach, the gene expression patterns of two training sample sets (for example, pathway 'on' and pathway 'off') are compared and differentially regulated genes are linearly combined into principal components, thereby reducing the dimensionality of the feature space. Binary regression on the principal components is then applied to an unknown test sample, resulting in a probability score describing the likelihood of pathway activation in that sample. This approach has several advantages. First, the output is, at least in theory, a quantitative measure of pathway activity. Furthermore, SVD regression can be applied to a single sample and does not require dividing the testing samples into two groups based upon *a priori *knowledge. Finally, the use of reduced-dimension features and orthogonal components reduces problems involving co-linearity during regression analysis. For these reasons, SVD regression holds promise as a mathematical tool for predicting pathway activity.

To date, SVD regression has been used to detect activation of dominant oncogenic signaling pathways, such as Myc or Ras, in MMTV-Myc and MMTV-Ras driven mouse breast cancer models, respectively [4,5,12]. In these contexts, SVD regression was shown to be capable of detecting activation of the pathway that was experimentally perturbed. While such experiments provided proof-of-principle that SVD regression can detect pathway activation, the critical question of whether SVD regression is sensitive enough to detect activation of endogenous pathways has not been fully addressed.

SVD regression has also been used to predict pathway activity in human samples [4,5]. For example, Bild *et al*. [4] were able to predict the activation status of five distinct oncogenic pathways (Myc, Ras, E2F, Src, and β-catenin) in primary lung cancers and to correlate these activities with patient survival. Unfortunately, validation of the sensitivity and specificity of this approach is limited by the difficulty in confirming predictions made on human samples, as material for biochemical analysis is often unavailable. Thus, the accuracy of predictions made using SVD regression in these studies remains undetermined.

We reasoned that SVD regression might be a powerful means of detecting endogenous pathway activation, allowing for the discovery of new biological relationships between signaling pathways. To evaluate this possibility, we addressed whether SVD regression is sufficiently sensitive to detect secondary activation of an endogenous pathway in a model amenable to experimental manipulation and validation. Specifically, we focused on the relationship between the Ras and transforming growth factor beta (TGFβ) signaling pathways. Although a number of studies have documented crosstalk between these pathways, a coherent model explaining their interaction has remained elusive, and there exists no consensus on the direction or underlying mechanism of this crosstalk, nor on how these pathways interact during epithelial cell transformation.

In non-transformed cells, the Ras and TGFβ pathways exert largely antagonistic effects: Ras can inhibit TGFβ-induced growth suppression by inhibiting Smad nuclear translocation [13], while TGFβ can potently inhibit cell proliferation induced by mitogenic factors, such as epidermal growth factor, that signal through Ras [14]. In contrast, Ras and TGFβ appear to cooperate in transformed cells to promote aspects of tumor progression, including epithelial-to-mesenchymal transition, invasion, and metastasis [15-17]. As such, crosstalk between the Ras and TGFβ pathways is complex, may occur at multiple nodes within each pathway, and is likely to be dependent upon cellular context.

To detect crosstalk between the Ras and TGFβ pathways using computational approaches, we generated gene expression signatures that allow for the quantitative prediction of TGFβ and Ras pathway activity using SVD regression. Using these signatures, we demonstrate that acute induction of oncogenic Ras in the mouse mammary gland results in rapid activation of the TGFβ pathway. Conversely, application of SVD regression using a Ras pathway signature revealed rapid Ras pathway activation following TGFβ treatment of normal mammary epithelial cells. Biochemical studies confirmed these computational findings, supporting the specificity of these SVD regression-based predictions. Taken together, our results indicate that SVD regression can detect activation of endogenous pathways *in vivo*, thereby providing novel insight into cell signaling *in vivo*.

## Results

### Generation of a TGFβ pathway signature using SVD regression

To generate a gene expression signature for the TGFβ signaling pathway in mammary epithelial cells, we used a non-transformed murine mammary epithelial cell line (NMuMG). NMuMG cells respond to TGFβ by undergoing epithelial-to-mesenchymal transition and have commonly been used to study signaling and transcriptional events downstream of this cytokine. To identify a comprehensive list of genes altered by TGF-β1 treatment, Affymetrix microarray analysis was performed on untreated NMuMG cells and cells treated with TGFβ for 24 h. SVD regression with Markov Chain Monte Carlo (MCMC) fitting generated a TGF-β1 signature consisting of 500 genes. Among the genes present in this signature were several known TGFβ targets, including *Serpine1*/*plasminogen activator inhibitor-1 *(*PAI-1*), *connective tissue growth factor *(*Ctgf*), *Bhlhb2*, *cysteine rich protein 61*(*Cyr61*) and *interleukin-11*(*IL-11*) [18-21].

**Figure 1:**
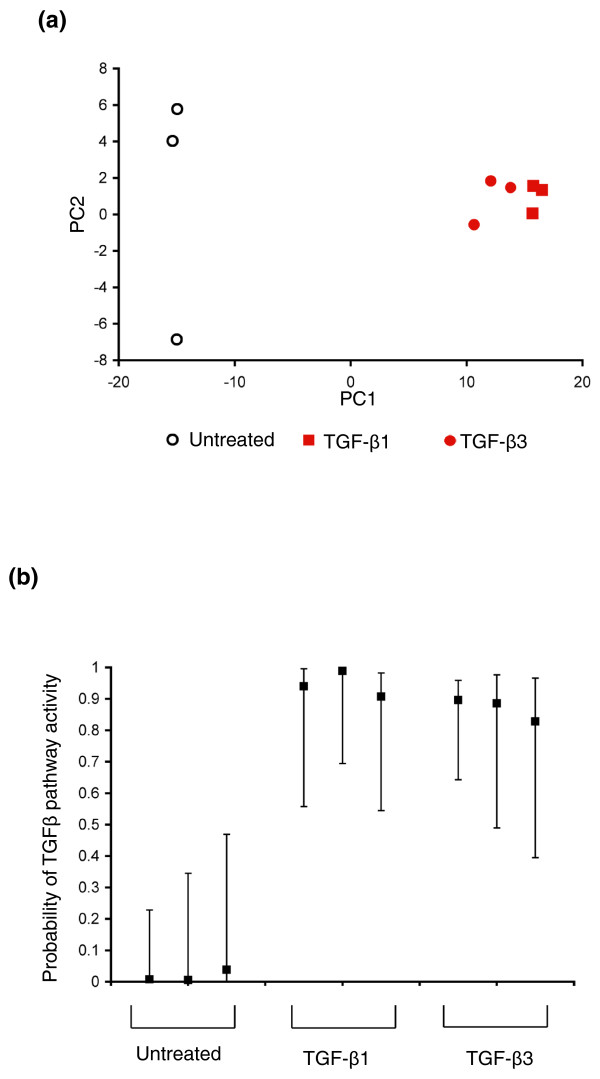
**An NMuMG-derived TGFβ signature accurately and quantitatively predicts TGFβ pathway activation**. **(a) **Principal component analysis (PCA) of untreated NMuMG cells (open circles), TGF-β1 treated cells (training set, filled squares), and TGF-β3 treated cells (testing set, filled circles). **(b) **SVD regression demonstrating quantitative prediction of TGFβ pathway activity in both TGF-β1 and TGF-β3 treated cells.

We next wished to compare the transcriptional changes induced by TGF-β1 and TGF-β3. NMuMG cells were treated with TGF-β3 for 24 h, Affymetrix microarray analysis was performed, and a TGF-β3 signature was extracted in a manner analogous to that used for TGF-β1. Principal component analysis (PCA) of the TGF-β1 signature revealed that 97.7% of the gene expression variation could be represented in principal component 1 (Figure 1a). When the TGF-β3 signature was projected in the PCA plot onto the space delineated by the TGF-β1 signature, TGF-β3-treated samples fell closer to TGF-β1 treated samples than to untreated NMuMG cells, indicating that TGF-β1 and TGF-β3 elicit similar transcriptional changes (Figure 1a).

To further compare the transcriptional changes induced by TGF-β1 and TGF-β3, the extent of overlap between genes differentially regulated by these cytokines was assessed. Treatment with TGF-β1 and TGF-β3 led to changes in 1,316 and 880 probes, respectively, with a minimum threshold of a 1.5-fold change and a *p*-value <0.01. There were 757 differentially regulated genes common to these two treatments (*p *= 1.2 × 10^-107^, hypergeometric test), indicating again that TGF-β1 and TGF-β3 induce very similar transcriptional programs. Since substantial overlap was identified between the TGF-β1 and TGF-β3 transcriptional responses, we used the 500-gene TGF-β1 signature as the TGFβ pathway signature for all subsequent experiments and the TGF-β3 dataset was used as an independent testing dataset (Additional data file 1).

### Quantitative estimation of TGFβ pathway activity in TGFβ-treated mammary epithelial cells using SVD regression

While PCA permits untreated and TGFβ-treated samples to be distinguished, it would be useful to have a quantitative measure of TGFβ pathway activity in a given sample. Given the limited sensitivity and specificity of microarrays [22-24], this requires combining multiple probe sets and reducing the dimensionality of data to construct a stable predictor with limited training data.

Toward this end, SVD binary regression with MCMC fitting was applied to obtain a quantitative measurement of TGFβ pathway activity. First, the TGFβ pathway predictor was trained by comparing TGF-β1 treated and untreated cells. The predictor was then tested on TGF-β3 treated cells. Using leave-one-out cross-validation to assess out-of-sample-error, the predictor was able to detect TGFβ pathway activity in both the training (TGFβ-1) and the testing (TGFβ-3) sets (Figure 1b). Thus, this model appears to provide a sensitive and accurate measure of TGFβ activity.

### PCA identifies TGFβ pathway activation following short-term Ras induction

Given the complex relationship between the Ras and TGFβ pathways during epithelial cell transformation [14-17,25-29], we sought to determine the status of the TGFβ pathway following Ras activation *in vivo*.

We previously described the generation of TetO-Ras (TRAS) mice in which expression of an activated oncogenic Ras allele (*Hras*^*G12V*^) is under the control of the tetracycline operator [30]. *TRAS *mice were mated to MMTV-rtTA (MTB) transgenic mice that express the reverse tetracycline transactivator (rtTA) under the control of the MMTV promoter. In the resulting bitransgenic MTB/TRAS mice, doxycycline treatment leads to oncogenic Ras expression in the mammary epithelium, resulting in the acute activation of pathways downstream of Ras [31].

**Figure 2:**
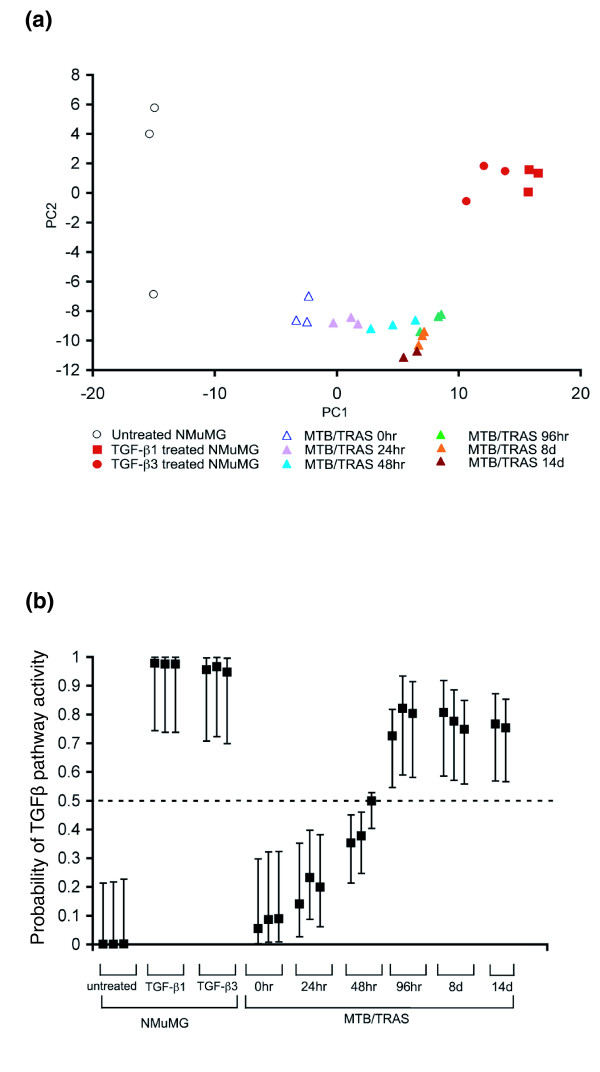
**A TGFβ signature detects TGFβ pathway activation following short-term Ras induction in the mammary gland**. **(a) **Mapping of mammary glands expressing activated Ras for increasing times (filled triangles) or uninduced controls (open triangles) onto the principal component space defined by the TGFβ signature in Figure 1a. **(b) **SVD regression predicts TGFβ pathway activation in mammary glands expressing activated Ras for 96 h, 8 days, and 14 days.

To examine the relationship between Ras activation and TGFβ pathway activity, we used microarray expression profiling and SVD regression to assess TGFβ pathway activity in the mammary glands of MTB/TRAS mice following doxycycline treatment. MTB/TRAS mice were treated with doxycycline for 24 h, 48 h, 96 h, 8 days or 14 days, and RNA was harvested from mammary glands for global gene expression analysis using Affymetrix microarrays. When mammary gland samples were projected onto the expression space delineated by the TGFβ signature, as defined in NMuMG cells, mammary samples in which Ras was acutely induced spanned the region between untreated and TGFβ-treated NMuMG cells (Figure 2a). Mammary gland samples from uninduced MTB/TRAS mice were most similar to untreated NMuMG cells, whereas mammary gland samples from 14-day induced MTB/TRAS mice were most similar to TGFβ-treated NMuMG cells. The magnitude of TGFβ activation predicted based upon the TGFβ signature increased with increasing duration of Ras activation. These results indicate that Ras activation in the mammary gland results in gene expression changes similar to those induced by TGFβ in mammary epithelial cells *in vitro*. This, in turn, suggests that oncogenic Ras is capable of directly activating the TGFβ pathway *in vivo*.

### SVD regression identifies TGFβ pathway activation following short-term Ras-induction

We next wished to obtain a quantitative measure of changes in TGFβ pathway activity following short-term Ras activation *in vivo*. To achieve this, the SVD predictor was used to estimate TGFβ activity at increasing times following Ras induction. This analysis revealed a time-dependent increase in predicted TGFβ activity in the mammary gland following Ras activation. An increased probability of TGFβ pathway activity was observed as early as 24-48 h following Ras activation. Increased TGFβ pathway activity reached statistical significance at 96 h post-Ras-induction and remained elevated through 14 days of Ras activation (Figure 2b). These results indicate that Ras activation in the mammary gland leads to the progressive, time-dependent induction of a TGFβ expression signature indicative of TGFβ pathway activity.

### Generation of a Ras pathway signature using SVD regression

**Figure 3:**
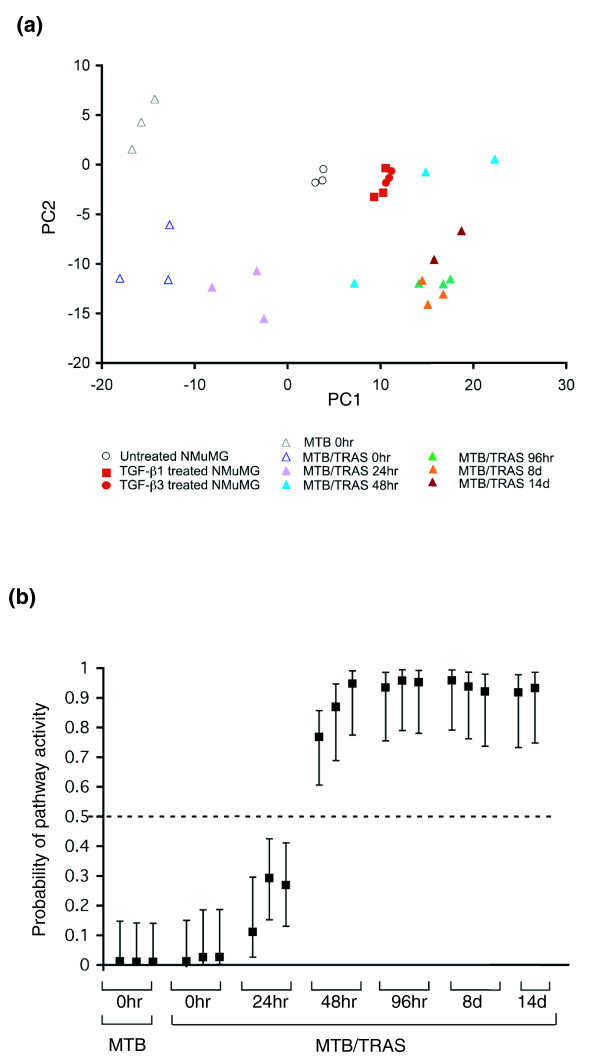
**An *in vivo*-derived Ras signature accurately and quantitatively predicts Ras pathway activation**. **(a) **PCA demonstrating separation of mammary gland samples with Ras activation (MTB/TRAS 48 h, 96 h, 8 days and 14 days, filled triangles) from uninduced controls (MTB and MTB/TRAS 0 hours, open triangles) across principal component 1 (PC1). MTB/TRAS mice uninduced (open triangles) or induced (filled triangles) for 48 or 96 h were used for training, while the remaining MTB/TRAS time points and MTB uninduced mice were used as the test set. **(b) **SVD regression demonstrating quantitative prediction of Ras pathway activation following short-term induction in the mammary gland.

We next sought to construct a predictor that would permit assessment of Ras pathway activity based on microarray data. To generate an *in vivo *Ras signature, SVD regression analysis with MCMC fitting was applied to expression data from the mammary glands of MTB/TRAS mice induced for 0, 48 or 96 h (Additional data file 2). When other induction time-points were projected onto this principal component space, early time-points (t = 24 h) fell closest to uninduced samples, whereas later time-points (t = 8 days and 14 days) fell closest to the 48 h and 96 h samples (Figure 3a). This indicates that the Ras signature generated from 48 h and 96 h induction time-points also detects Ras activity following earlier as well as later times of induction, thereby validating the utility of this signature.

To obtain a quantitative measure of Ras pathway activity, SVD binary regression was applied to expression data from MTB/TRAS mice induced for 0, 48 or 96 h. The resulting predictor was then applied to the other induction time-points to test its ability to quantitatively predict Ras activity. MTB/TRAS mice induced for 24 h exhibited a detectable increase in Ras pathway activity that was higher than that observed for MTB controls and lower than that observed for MTB/TRAS mice induced for 48 h (Figure 3b). MTB/TRAS mice in which Ras was induced for 8 or 14 days displayed pathway activation higher than that observed at 48 h and comparable to that observed following 96 h of Ras transgene induction (Figure 3b). These findings indicate that this gene predictor accurately and quantitatively detects Ras pathway activation.

### SVD regression identifies endogenous Ras pathway activation following TGFβ treatment

In light of our computational prediction that acute Ras activation in the mammary gland resulted in secondary activation of the TGFβ pathway, and in light of prior reports implicating the mitogen-activated protein kinase (MAPK) pathway in TGFβ-induced epithelial-to-mesenchymal transition [32], we sought to determine whether acute TGFβ pathway activation in mammary epithelial cells resulted in secondary activation of the Ras pathway. First, gene expression data from untreated, and TGF-β1- and TGF-β3-treated NMuMG cells were mapped onto the principal component space defined by the *in vivo *Ras signature. TGF-β1- and TGF-β3-treated cells mapped closest to the 8- and 14-day Ras-induction samples, whereas untreated cells mapped closer to uninduced samples (Figure 3a) This suggests that TGF-β1 and TGF-β3 induce transcriptional changes similar to those induced by Ras activation.

**Figure 4:**
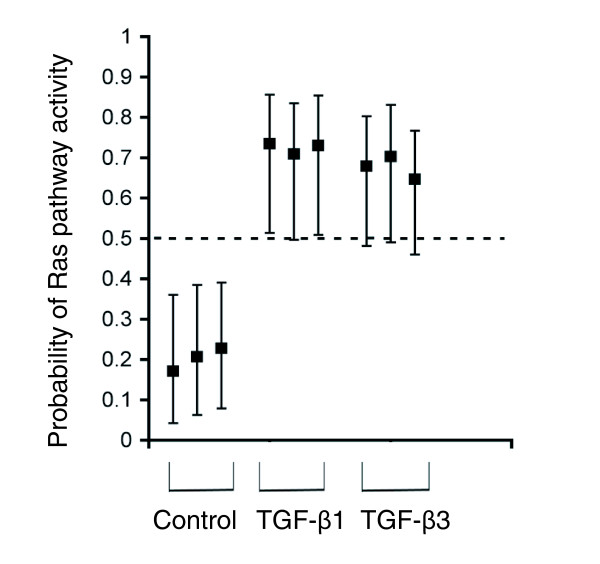
**A Ras signature detects Ras pathway activation following TGFβ treatment of NMuMG cells**. SVD regression predicting activation of the Ras pathway in TGF-β1- and TGF-β3-treated NMuMG cells, but not untreated controls.

To quantitatively assess the level of Ras pathway activation induced by TGFβ treatment, the Ras predictor was applied to TGF-β1- and TGF-β3-treated NMuMG cells. Whereas untreated NMuMG cells displayed no detectable increase in Ras pathway activity, TGF-β1 and TGF-β3 treatment led to the robust induction of signatures indicative of Ras pathway activation (Figure 4). Together, both PCA and SVD regression analyses predict that the Ras pathway is activated as a consequence of TGFβ treatment in NMuMG cells.

### Biochemical validation of pathway predictions

We considered several models to explain the pathway predictions made by SVD. First, Ras and TGFβ might initiate similar gene expression programs through distinct transcriptional mediators. Alternatively, Ras might lead to activation of regulatory molecules downstream of TGFβ, such as those of the Smad transcription factor family. Similarly, TGFβ might activate effector molecules downstream of Ras, such as Raf, MEK, and MAPK. To evaluate these possibilities at the biochemical level, we examined the Smad family of transcription factors as well as the Raf-MEK-MAPK pathway as critical mediators of TGFβ and Ras-induced signaling, respectively.

**Figure 5:**
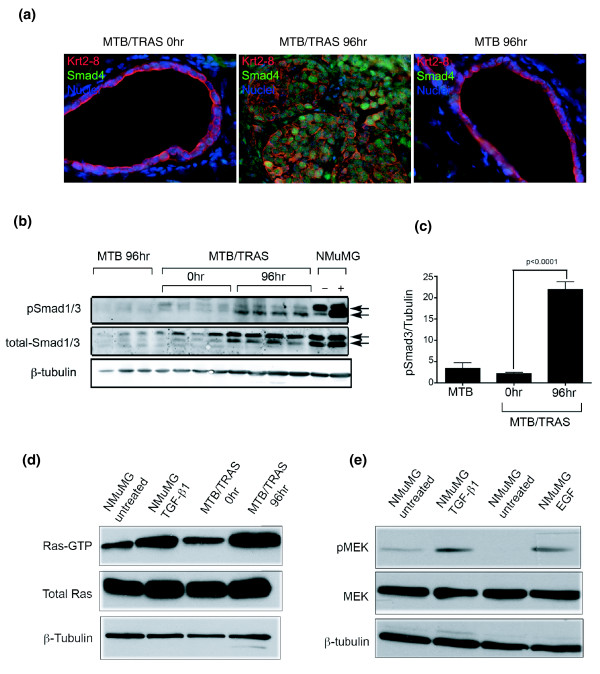
**Ras and TGFβ exhibit positive reciprocal regulation in mammary epithelial cells**. **(a) **Immunofluorescence showing Smad4 nuclear translocation following short-term Ras expression in the mammary gland. Nuclei (blue), Smad4 (green), cytokeratin 8 (red). **(b) **Western blot analysis demonstrating phosphorylation of Smad1/3 after 96 h of Ras activation *in vivo*. **(c) **Quantification of western analysis. **(d) **Western analysis showing activated, GTP-bound Ras in NMuMG cells following TGFβ treatment. **(e) **Western analysis showing activated MEK in NMuMG cells following TGFβ treatment.

To determine whether the activation of the TGFβ pathway that we detected computationally following short-term Ras induction in the mammary gland was due to activation of Smad transcription factors, we performed immunofluorescence on mammary gland sections to examine the subcellular localization of Smad4. This analysis revealed that 96 h of Ras activation in the mammary gland was sufficient to induce nuclear translocation of Smad4, confirming activation of this pathway (Figure 5a). We next examined Smad3 phosphorylation following Ras activation. Consistent with our prediction that Ras activates this pathway, we found that acute induction of activated Ras led to a marked increase in levels of phosphorylated Smad3 (Figure 5b,c). Thus, short-term Ras activation directly induces Smad activation *in vivo*, which in turn results in the induction of a TGFβ transcriptional response.

To test our prediction that TGFβ treatment results in Ras pathway activation, the activation status of signaling components of this pathway was evaluated in TGFβ-treated NMuMG cells. As predicted, levels of Ras-GTP were higher in TGFβ-treated NMuMG cells compared to untreated cells (Figure 5d), indicating that TGFβ treatment resulted in Ras activation. Similarly, while TGFβ treatment did not alter the activation of RalA/B or Akt in NMuMG cells (data not shown), significant increases in p-MEK levels were observed in NMuMG cells following TGFβ treatment (Figure 5e). This indicates that TGFβ treatment results in Ras-Raf-MAPK pathway activation in NMuMG cells *in vitro*, thereby confirming our computational prediction.

Together, our results are consistent with a model in which oncogenic Ras activation results in the induction of a TGFβ transcriptional response through activation of Smads, and in which activation of the TGFβ pathway can induce a Ras transcriptional response by activating the Ras-Raf-MAPK pathway.

### SVD regression identifies TGFβ pathway activation in Ras-induced mammary tumors

**Figure 6:**
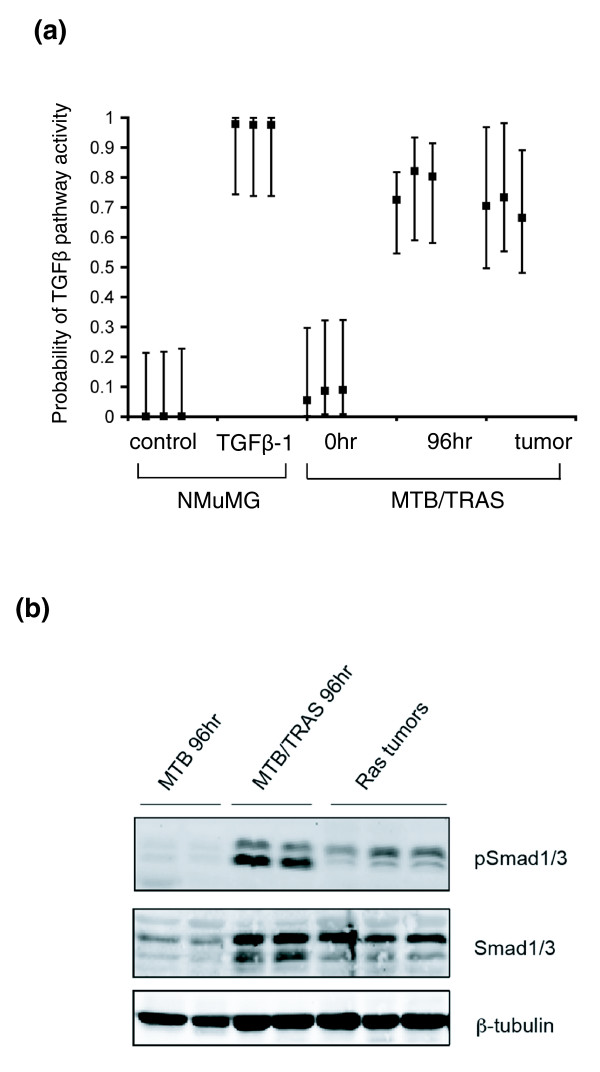
**A TGFβ signature detects TGFβ pathway activation in Ras-induced mammary tumors**. **(a) **SVD regression predicts TGFβ pathway activation in mammary glands expressing activated Ras for 96 h and in mammary tumors induced by chronic Ras activation. **(b) **Western analysis showing increased phosphorylation of Smad1/3 in Ras-induced mammary tumors.

The results described above indicate that SVD regression can detect endogenous activation of a secondary pathway in a well-defined system. For SVD regression to be of broad utility, however, it must also accurately predict pathway activation in a complex system, such as a tumor. Chronic Ras activation in the mammary gland leads to the formation of adenocarcinomas with a latency of 14 weeks. Given our finding that short-term Ras activation in the mammary gland results in TGFβ pathway activation, we next sought to assess whether activation of the TGFβ pathway is also detectable in Ras-induced tumors. To address this, global gene expression profiles of Ras-induced tumors were assessed by Affymetrix microarray analysis, and the above SVD predictor was used to predict their TGFβ pathway activity. This analysis reveals that the TGFβ pathway is indeed activated in Ras-induced tumors (Figure 6a), suggesting that this putative tumor suppressor TGFβ pathway is not shut off during the course of Ras-induce tumorigenesis.

We next used biochemical approaches to test our computational prediction that the TGFβ pathway is activated in Ras-induced tumors. Lysates from Ras-induced tumors were prepared and levels of activated Smad1/3 were assessed by western blot. We observed prominent Smad1/3 phosphorylation in Ras-induced mammary tumors (Figure 6b), confirming our computational prediction that the TGFβ pathway remains activated in Ras-induced tumors. This indicates that SVD can detect signaling pathway activation within a complex system.

## Discussion

The ability to detect activation of an oncogenic pathway based upon patterns of gene expression would constitute a useful tool to query tumor biology and aid in prognostic and therapeutic decision-making in cancer patients. Herein we describe the use of SVD regression to accurately detect endogenous pathway activity *in vivo *in the context of a strong primary oncogenic stimulus. Using an inducible transgenic model expressing oncogenic Ras in the mammary gland, we have demonstrated that a TGFβ transcriptional signature is induced following short-term Ras activation and remains elevated during a 2-week course of Ras induction in the mammary gland. We have further demonstrated that this signature can be attributed to Ras-induced activation of Smad transcription factors, which provides a mechanistic basis for our computational prediction. Finally, we have demonstrated that TGFβ treatment of NMuMG cells results in the rapid induction of a Ras pathway signature. Consistent with these computational predictions, biochemical studies revealed that TGFβ treatment resulted in MEK activation and increased levels of Ras-GTP, suggesting that induction of the Ras-MEK-ERK pathway is responsible for induction of the observed Ras signature following TGFβ treatment.

Taken together, our results suggest a model in which Ras and TGFβ induce reciprocal positive crosstalk in non-transformed mammary epithelial cells. Since TGFβ has been shown to inhibit epithelial cell transformation [33], our finding that TGFβ activity is increased following activated Ras expression in the mammary gland was unexpected, given that Ras induces widespread hyperplasia in the mammary gland at the time points tested and ultimately leads to tumor formation. However, these results are consistent with reports that Ras and TGFβ can synergize in promoting some aspects of the malignant phenotype [15,17]. Our findings provide important confirmation of this hypothesis in an *in vivo *model for mammary tumorigenesis and suggest that, at least in the context of Ras activation, the TGFβ pathway could potentially contribute to early stages of transformation.

Using gene expression patterns to predict pathway activity has several advantages over traditional biochemical methods. Such signatures are based upon downstream transcriptional targets of a pathway, and so function as an overall measure of pathway activity. In contrast, biochemical approaches generally focus on one or several nodes in a pathway. Consequently, these approaches risk missing pathway activation that occurs at other points in the pathway, or that results from subtle, coordinated changes in multiple pathway members. While computational prediction of pathway activity does not address the mechanism by which a given pathway is activated, it does generate testable predictions for subsequent experiments.

Although linear regression is a popular tool in prediction, we did not use it here to predict pathway activity for two reasons. First, our training dataset only has two states, pathway 'on' and 'off', and linear regression is not suitable in such cases. Second, the number of training samples is much smaller than the number of signature genes, a problem known as the 'curse of dimensionality' in statistical learning. This makes estimation of the linear regression coefficient unstable. To circumvent this problem, SVD has been used for dimensionality reduction. For instance, SVD has been used to reduce the dimensionality of expression data and integrate ChIP-chip data with expression data [34,35]. It has also been employed to reduce the expression data dimension prior to classifier training using support vector machines [36,37]. Although each of these approaches used SVD to reduce dimensionality, the objectives of these studies were distinct from those of this study, which focused on using expression data to predict signaling pathway activity.

Until recently, SVD binary regression has primarily been used to detect the activity of ectopically activated dominant oncogenic pathways [4,12]. Whether it can also be used to detect endogenously occurring activation of a secondary pathway had not previously been assessed. We were able to detect TGFβ pathway activity in the context of concurrent, strong Ras pathway activation, and vice versa. Our findings, which were unexpected, indicate that SVD regression can detect crosstalk between endogenous signaling pathways and may be useful for identifying previously unsuspected relationships between signaling pathways. Furthermore, our results provide an important proof-of-principle that SVD regression is sufficiently sensitive for this purpose, which is essential for the utility of this technique in predicting pathway activity in human cancers.

When analyzing gene expression data from human tumor samples, lack of materials frequently renders biochemical validation impossible. As such, validating signatures in experimentally tractable systems is valuable. To this end, in the study presented here we were able to validate our computational predictions with biochemical approaches. Given that tumors typically result from the collaboration between multiple signaling pathways, the ability to detect the activation status of individual pathways within a complex network of other pathways in the cell is of paramount importance. In this manner, it should be possible to classify tumors according to the molecular pathways that have been activated, thereby leading to improvements in the selection of appropriate treatments.

## Materials and methods

### Inducible transgenic mice and cell culture

MTB and TRAS transgenic mice have previously been described [30,38]. Bitransgenic MTB/TRAS mice in an FVB/N background were generated by crossing MTB and TRAS mice. To induce oncogenic v-*H-Ras *expression, 6-week-old MTB/TRAS female mice were administered 2 mg/ml doxycycline with 5% sucrose in their drinking water. Mammary tissue was harvested at different post-induction time points and snap frozen. To generate Ras-driven tumors, MTB/TRAS mice were administered 0.012 mg/ml doxycycline in their drinking water and monitored for tumor formation. Mice were sacrificed when tumors reached approximately 1 cm and tissue was snap frozen.

The non-transformed murine mammary epithelial cell line NMuMG was cultured in Dulbecco's modified Eagle's medium (DMEM) supplemented with 10% bovine calf serum, 1% penicillin/streptomycin, and 2 mM L-glutamine. For TGFβ treatment, cells were cultured in low serum medium (0.5%) overnight followed by treatment with 5 ng/ml TGF-β1 or TGF-β3 (Sigma, St. Louis, MO, USA). After 24 h, RNA and protein were harvested for microarray hybridization or biochemical analysis.

### Microarray analysis

RNA was isolated from snap-frozen mammary tissue or NMuMG cells as previously described [39]. The synthesis of biotinylated cRNA and hybridization to high-density Affymetrix MG-U74Av2 microarrays were performed according to manufacturer's instructions. The raw data can be accessed through the GEO database [GEO:GSE13986]. Genechip Robust Multichip Average (GCRMA) was used to extract signal values from CEL files [22,24]. Expression values were log2 transformed. The arrays were normalized using quantile normalization and a fold-change based filtration was applied to all genes on the array. Genes whose expression changed by less than 1.5-fold between the two perturbed states were filtered out as non-changing genes.

### SVD binary regression

The method we used for pathway activity prediction uses a standard binary regression model in combination with SVDs. Suppose a binary phenotype, such as disease class, and expression levels for *p *genes are collected on *n *independent samples. The *n *× 1 response vector *y *and the *p *× *n *gene expression matrix *X *can be related using the probit regression model, *E [Y] *= Φ(*X' β*), where *Φ *is the cumulative distribution function of the standard normal distribution. In microarray studies, we usually have *p *>> *n *and this makes inference of the regression coefficients, *β*, unstable. To circumvent this problem, a SVD is applied to *X*, *X *= *ADF*. The probit model can then be written as *E [Y] *= Φ(*F'DA' β*) = Φ(*F' θ*), where *F *is *n *× *n *matrix of metagenes and *θ *= *DA'β*. SVD therefore reduces the dimensionality of the parameter space. The parameter estimation on θ is implemented using MCMC simulation methods and Bayesian inference [7]. The software is implemented in Matlab and is available for download [12].

### Pathway signature analysis

To construct a pathway activity predictor for TGFβ, we first performed a 1.5-fold change based filtration on TGFβ1-treated versus untreated NMuMG microarray data. To obtain a TGFβ pathway predictor, we trained SVD binary regression using the differentially regulated genes. The parameters that were used to train SVD binary regression were chosen according to described guidelines [4]. For the MCMC procedure, we used 5,000 iterations for burn-in and 5,000 iterations to estimate regression coefficients. To predict TGFβ pathway activity on a new sample, we used the learned parameters to project that sample onto the principal component space and computed the probability of pathway activation. The same parameters were used to construct a Ras pathway predictor. The genes that are in common between TGFβ and Ras pathway signatures are listed in Additional data file 3.

### Immunofluorescence analysis

Mammary tissues embedded in Optimal cutting temperature compound (OCT) (Torrance, CA, USA) were sectioned at 8 μm and fixed for 10 minutes in 4% neutral buffered paraformaldehyde. Following three 10-minute rinses in phosphate-buffered saline (PBS), antigen retrieval was performed by heating sections in pH 6.0 citrate buffer. Sections were then rinsed in PBS and incubated in blocking buffer (5% bovine serum albumin, 0.3% Triton X-100, 10% normal goat serum, in PBS) for 1.5 h at ambient temperature. Primary antibodies diluted in blocking buffer were applied to each section and incubated at 4°C overnight. Unbound primary antibody was removed with three 10-minute rinses in wash buffer (0.3% Triton X-100 in PBS), and sections were subsequently stained with Alexa Fluor^® ^488 or 567 conjugated goat IgG serum raised against the host of the primary antibodies (Molecular Probes, Carlsbad, CA, USA). Stained sections were rinsed for 10 minutes in wash buffer and twice for 10 minutes each in PBS. Nuclei were counterstained with 1 μg/ml Hoechst 33258 dye, mounted in Fluoromount-G (SouthernBiotech, Birmingham, AL, USA), and visualized using a Leica DMRXE microscope.

### Immunoprecipitation and western blot analysis

Tissue lysates were prepared from snap frozen mammary tissues or NMuMG cells by Dounce homogenization using a magnesium lysis buffer (Upstate Biologicals, Billerica, MA, USA). The levels of Ras-GTP or RalA/B-GTP were detected using Ras and RalA activation kits (Upstate Biologicals) according to the manufacturer's instructions. Western blot analysis was performed as described [40]. The following primary antibodies were used for western blot analysis: anti-phospho-MEK1/2 (Ser217/221; Cell Signaling, Danvers, MA, USA); anti-phospho-Smad1/3 (Ser423/425; Cell Signaling); anti-Smad3 (Santa Cruz, CA, USA); anti-phospho-Akt (Ser437; Cell Signaling); anti-Akt (Cell Signaling); and anti-β-tubulin (Biogenex, San Ramon, CA, USA). Secondary antibodies were horseradish peroxidase-conjugated goat anti-mouse and horseradish peroxidase-conjugated goat anti-rabbit antibodies (Jackson ImmunoResearch, West Grove, PA, USA). All primary antibodies were incubated at 4°C overnight. Secondary antibodies were incubated for 1 h at room temperature.

## Abbreviations

GSEA: gene set enrichment analysis; MAPK: mitogen-activated protein kinase; MCMC: Markov Chain Monte Carlo; PBS: phosphate-buffered saline; PCA: principal component analysis; SVD: singular value decomposition; TGFβ: transforming growth factor beta.

## Authors' contributions

ZL, MGT, and LAC conceived the study. ZL and TCP performed the computational studies. MW, JVA, MEB, and CCC carried out the biochemical validation experiments. ZL, MW, JVA, CD, MGT, and LAC drafted the manuscript. All authors read and approved the final manuscript.

## Additional data files

The following additional data are available with the online version of this paper. Additional data file 1 is a spreadsheet of the gene signature for TGFβ pathway, including probe set ID, log fold change, gene name, Entrez ID, and gene symbol. Additional data file 2 is a spreadsheet of the Gene signature for Ras pathway, including probe set ID, log fold change, gene name, Entrez ID, and gene symbol. Additional data file 3 is a spreadsheet of the genes in common between TGFβ signature and Ras signature.

## Supplementary Material

Additional data file 1 Gene signature for TGFβ pathway, including probe set ID, log fold change, gene name, Entrez ID, and gene symbol. Click here for file

Additional data file 2 Gene signature for Ras pathway, including probe set ID, log fold change, gene name, Entrez ID, and gene symbol. Click here for file

Additional data file 3 Genes in common between TGFβ signature and Ras signature. Click here for file
